# Biocementation beyond the Petri dish, scaling up to 900 L batches and a meter-scale column

**DOI:** 10.1038/s41598-025-87074-9

**Published:** 2025-01-24

**Authors:** Dimitrios Terzis, Camilla Perego, Margherita Cappa, Elisa Pianta, Federica Mauri, Pamela Principi

**Affiliations:** 1 Faculty of Environment, Architecture and Civil Engineering (ENAC), Swiss Federal Institute of Technology, Lausanne (EPFL), Lausanne, Switzerland; 2 Environmental Biotechnology, Institute of Microbiology, Department of Environment, Construction and Design, University of Applied Sciences and Arts of Southern Switzerland (SUPSI), 3 Medusoil SA, EPFL Innovation Park Building A, Manno, Switzerland; 3 Medusoil SA, EPFL Innovation Park Building A, Lausanne, 1015 Switzerland

**Keywords:** Biocementation, Ureolysis, MICP, Bioreactor, Cultivation, Mineralization, Sustainability, Environmental microbiology, Environmental impact, Civil engineering, Biogeochemistry

## Abstract

Microbial-induced calcite precipitation (MICP), which leverages ureolytic microorganisms, has received significant attention during the past decade as a promising method for sustainable building and geoenvironmental applications. However, transitioning from lab-scale experimentation to volumes suitable for practical use poses challenges. This study addresses these obstacles by screening and analyzing over 50 strains sourced from (i) a natural environment in the canton of Ticino in Switzerland; (ii) microorganism banks; and (iii) an industry-scale bioreactor. Several ureolytic *Sporosarcina* species have been identified in the natural environment, and their ureolytic potential has been compared with that of other strains. A reference, banked microorganism yielded the highest ureolysis rate. When this latter strain was inoculated in 900 L batches and continuously cultivated at 5400 L, no contamination issues were observed, and the reference strain remained the dominant species. The produced culture, obtained under an optimized medium composition involving the circular valorization of NH_4_^+^, was subsequently used to induce the biocementation of a 650 kg column of 0–1 mm sand. The results reveal the successful stabilization of the whole mass, with undrained Tresca strength values ranging from 90 to 140 kPa. This research lays the groundwork for scalable MICP production, which is capable of meeting the demands of real-world building and geoenvironmental projects.

**Supplementary Information:**

The online version contains supplementary material available at 10.1038/s41598-025-87074-9.

## Introduction

There is extensive knowledge concerning the conditions and substrates that favor the growth of ureolytic strains and the subsequent precipitation of CaCO_3_ minerals. This process, widely known as Microbial-induced calcite precipitation MICP, results in the formation of mineral links that act as binders and strengthen the properties of soil aggregates^1–4^. These binders have been described in the literature as sustainable alternatives to commonly applied binders such as lime^5^ or cementitious grouts^6,7^, which are known to induce highly alkaline environments. Other binders, such as polyurethane resins and foams result in the release of potentially harmful substances upon degradation^8^.

**Fig. 1:**
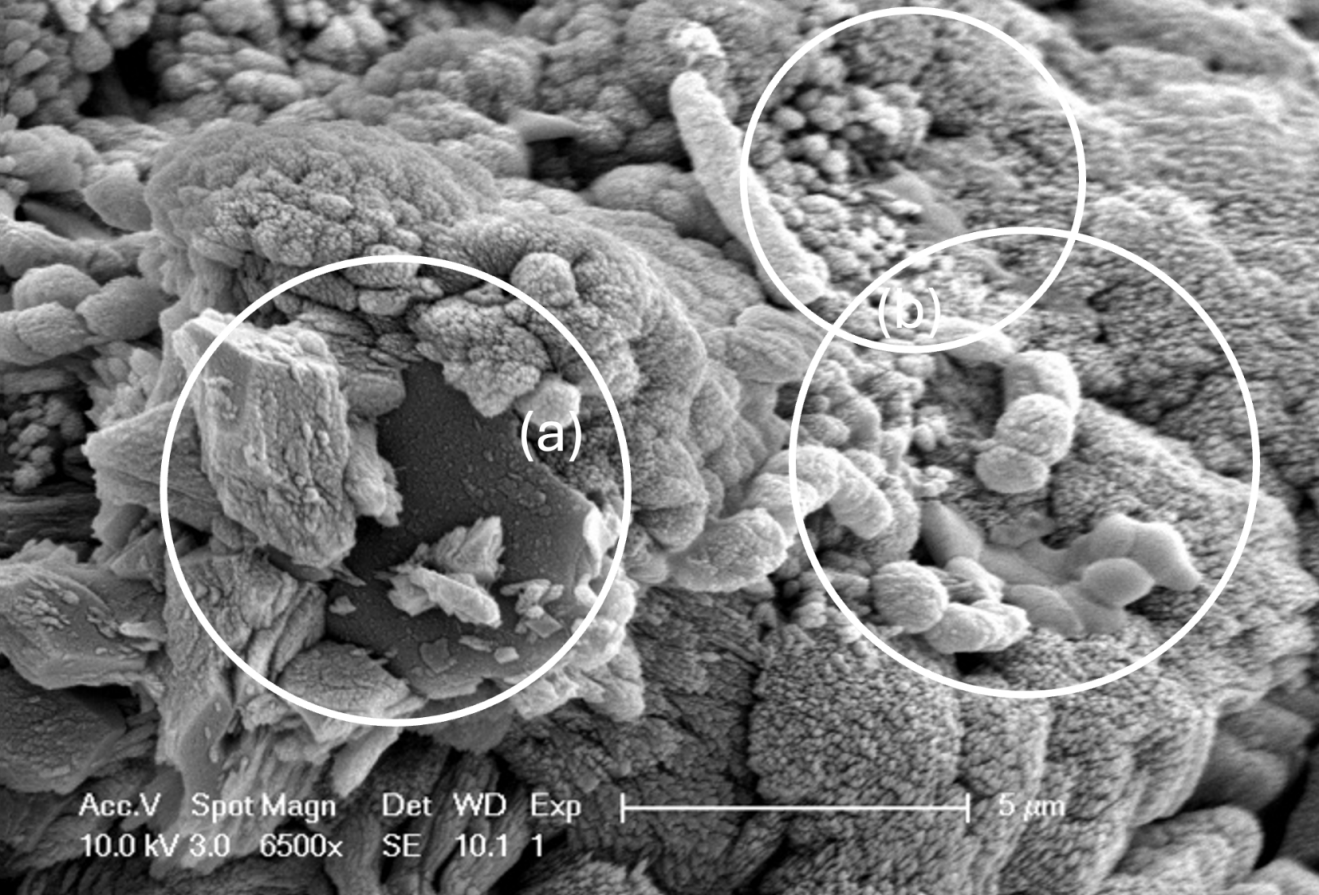
CaCO_3_ crystal precipitation and S. pasteurii cells. Inlet (**a**) shows the typical calcite phase with distinctive planar and cubic textural features; Inlet (**b**) illustrates epicellular precipitation of CaCO_3_ on rod-shaped cells of S. pasteurii, which remain encapsulated and hence are considered inactive, as they are deprived of nutrients.

MICP has been reported in several applications in the fields of geotechnical engineering^9^ and geoenvironmental work with a focus on erosion^10^, architectural structures and monuments^11,12^, carbon sequestration^13^ or even heavy metal and contaminant immobilization ^14,15^ and space exploration conceptual studies^16^. Notably, improvements in the mechanical^17^ or thermal properties^18^ do not compensate for the soil permeability because the pore space remains largely open^17,19^. A common denominator in all these works is the relatively attractive idea of using micrometer-scale agents, i.e., microorganisms that are native to various hydrogeological environments, and provide suitable conditions to induce the precipitation of CaCO_3_ in pH environments that are compatible with those of soils during a reaction which lasts several hours^20^. Among the most predominant ureolytic strains used are *Sporosarcina pasteurii*^21^ (Fig. 1), *Sporosarcina aquimarina*^22^, *Sporosarcina ureae*^23^, and *Bacillus subtilis*^24^.

Typical media used for growth include yeast extract, Tris-buffer and ammonium sulfate (ATCC 11859^21^. Urea, nutrient broth and calcium chloride are widely used to induce CaCO_3_ precipitation in environments where microorganisms have grown at sufficient biomass levels and have ideally attached to the aggregate surface to provide nucleation sites (Fig. 1). With the exception of urea and calcium chloride, which are widely available and find applications in agriculture or other industrial processes, the remaining components have considerable supply and economic constraints, which raises questions around the application of MICP at realistic scales. Even if alternative nutrients are to be used, further challenges related to maintaining conditions that enable the growth of ureolytic strains in large-volume reactors, including temperature, the aeriation ratio^25^, and competition with other species^26^, are needed. For such large-scale volumes, sterile conditions cannot be easily ensured without sophisticated equipment. This would imply intermediary steps and longer timeframes of potential MICP applications in a field, i.e., building works, that favor fast, relatively simple, and straightforward steps of execution.

To the best of the authors’ knowledge, minimal information is known^27,28^, about the continuous growth of microbial cultures in the order of thousands of litters used to induce MICP while addressing the problem of contamination and the quantification of the complete timeframe, from the inoculation of bacterial batches to the removal of unwanted nitrogen byproducts.

Based on the above information, this work aims to address the following considerations. If MICP aims to harness naturally occurring microorganisms, they must be sourced from natural environments. To do so, this work compares the performance of naturally isolated strains to those sourced from microorganism banks. How can one subsequently grow sufficient biomass to induce MICP using solutions on the order of thousands of liters that would be compatible with real-scale applications? This growth should occur while maintaining noncontaminated conditions and avoiding costly bench-scale procedures, which might hinder the scalability of the technique (sterile conditions, energy-intensive steps and high-purity nutrients). For this purpose, several batches of 900 L each were grown continuously, and the resulting culture was subsequently inoculated in a 1.5-meter column composed of fine aggregates. This setup aims to provide a reference example at a scale that can be considered representative of real building applications.

## Materials and methods

The most widely used strain in the laboratory on the bench scale is *Sporosarcina pasteurii*^21^, which was used in this study as the reference (wild type). Another strain with known ureolytic potential was supplied by the Culture Collection of Switzerland under the reference name nhaC (CCOS 1977) courtesy of Medusoil SA. Furthermore, the continuous growth of this latter strain under nonsterile conditions in 2 L and 1000 L reactors resulted in four additional strains. These isolates, namely, b1 and b2 from the 2 L reactors and p1 and p3 from the 900 L reactors, were used in this study and were compared with the performance of the banked microorganisms. The cultivation protocol is described in the Materials and Methods section. In addition to these six cultures, we further assessed more than fifty morphologically different strains isolated from ammonia-rich soil. All the isolates were screened for the presence of urease by growth on urea agar base plates, and those that yielded positive results were further analyzed according to the steps described below.

### Screening modality

The isolates from the environment, nonsterile reactors and reference cultures were subsequently screened for their safety to filter out potentially pathogenic strains and for their urease activity. For safety concerns, the isolates characterized using MALDI-TOF as belonging to the *Bacillus cereus* group were further screened using PCR for the presence of genes encoding the enteroxins hemolysin BL, nonhemolytic enterotoxin, cytotoxin K and cereulide, namely, hbl, nhe, cytK and ces, respectively. The presence of one of these genes was considered the cause of exclusion. The excluded strains were not further processed (i.e., their urease activity data are not available in Table 1).

### Urease activity

The isolates that passed the safety test were screened for urease activity, as urease activity is the primary indicator of the performance of the strain with respect to MICP applications. This is due to the fact that ureolysis is the first step in the MICP process and metabolic pathway, which ultimately leads to calcium carbonate precipitation.

### Samples and sampling

In April 2023, a total of 4 soil samples (10 g each) were collected from an agricultural area known to be exposed to high urea concentrations due to animal activity in the Ticino region in Switzerland (45°51’02.5’’N, 8°59’48.5’’). Samples from reactors inoculated with booster substrate and 900 L of bioreactor substrate were taken.

### Isolation and screening for urease activity

The isolation procedure for the environmental samples was performed following the methodology described in ^29^. This involved suspending a known quantity of soil in enrichment broth and sequentially isolating it on a urea-based agar plate (urea agar (Base) acc. CHRISTENSEN, Millipore, Buchs, Switzerland). The colonies that successfully changed color to pinkish-red due to an increase in pH associated with urease activity were then further investigated. The liquid samples were directly isolated on a urea-based agar plate.

The occurrence of urea hydrolysis during the growth phase of the isolates was determined based on conductivity measurements over time in triplicate for each strain. In fact, the hydrolysis of urea releases ammonia ions, increasing the conductivity of the solution. The theoretical value of mS/cm calculated from the urea concentration^30^ is considered the theoretical maximum value as per the relationship: Urea hydrolyzed (mM) = Conductivity variation (mS/cm) x 11.11.

**Fig. 2:**
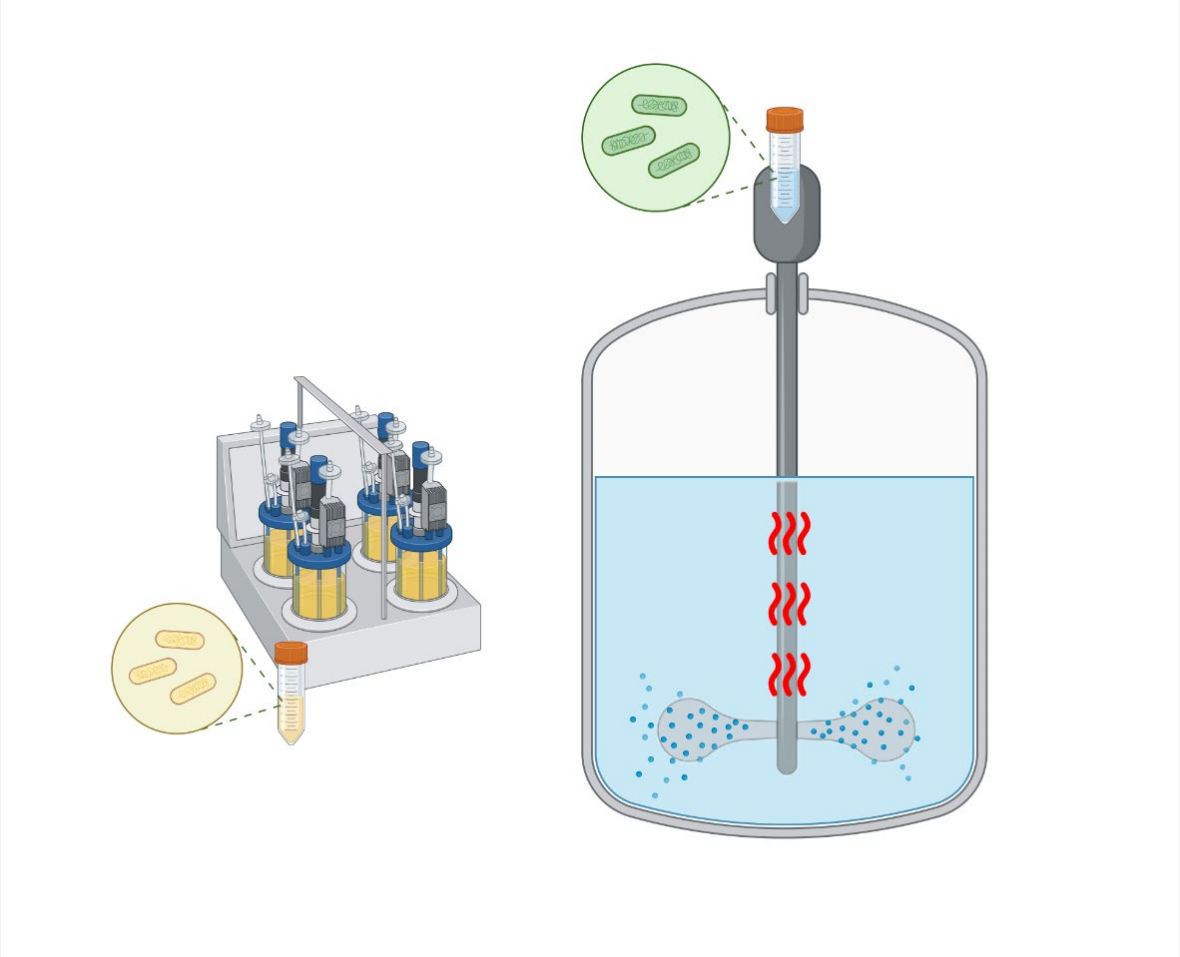
Schematic representation of the 2-step batch growth of S. pasteurii, including (**a**) an initial inoculum of 8 L where the strains were sampled and (**b**) a 1000 L reactor, with air supplied as the means of agitation and aeration as well as constant temperature maintenance at 30 °C. Created in BioRender. Terzis, D. (2024) https://BioRender.com/k66m363.

A 50 ml inoculum of SP_nhca was inoculated into each of the four 2 L containers, which were supplied at constant temperature using a heating plate together with agitation at 2000 rpm, as shown in Fig. 2. The strain grows in 10 g/L peptone and 20 g/L urea media, which represents a notable shift from bench-scale protocols that mobilize yeast extract, Trizma base and ammonium sulfate. A total of 8 L cultivated for 48 h was subsequently used as inoculum in 1000 L containers. The culture-to-air ratio was 9/10 which explains why a 1000 L container receives a 900 L liquid culture. An air pump is constantly used as a means of agitation and aeration at the bottom of the container. Samples were collected at various times to assess the evolution of hydrolysis using electrical conductivity measurements.

### Sand column production

Infiltration occurred from the bottom of the column via a 25 mm thick tube and progressed toward the top of the column. The bacteria from batch #6 were left for 12 h to attach before an equimolar solution of urea-CaCl_2_ (1 M) was introduced. An extraction pump was used to direct the effluent solution toward the tube again for a total of 24 h in 3 daily steps of 8 h each. After the last cycle, the extracted ammonium-rich solution was treated following the process described in^31^ to revalorize ammonium into ammonium sulfate, a fertilizer extensively used in agriculture.

## Results

### MICP strain collection

**Fig. 3:**
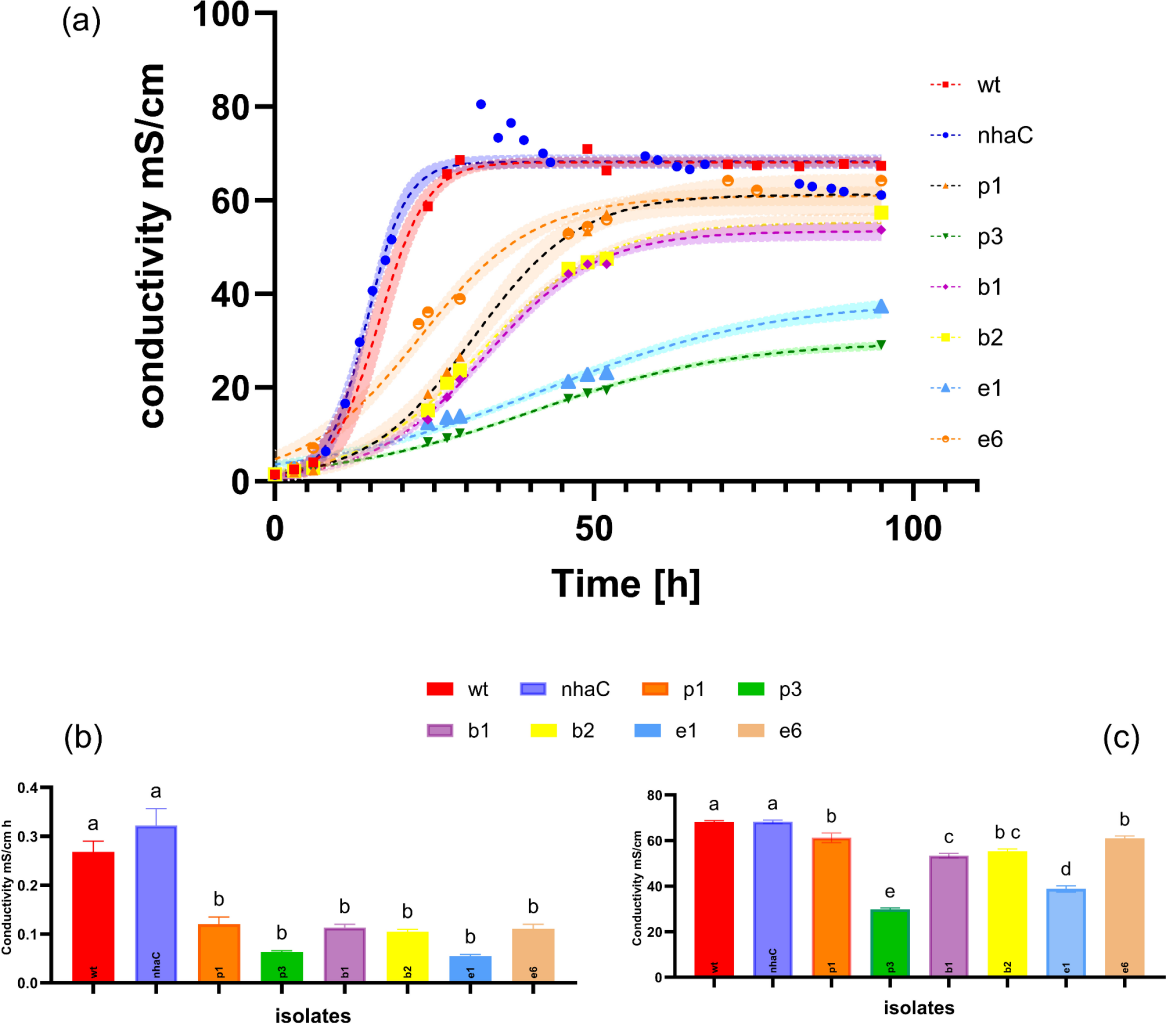
Bench-scale analysis of the ureolytic potential of eight strains. (**a**) EC monitoring and kinetic parameter assessment, including (**b**) the maximum slope of the curve (r_m_) and (**c**) the maximum value at plateau (y_m_). The same letters in the conductivity kinetics columns indicate significant differences at *P* < 0.05 according to one-way ANOVA and Tukey’s post hoc analyses.

Several sources for obtaining ureolytic microorganisms have been used. Specifically, these sources include (i) strains with known ureolytic potential for application of MICP supplied from two microorganism banks; (ii) the strains resulting from enrichment in 900 L reactors and the booster solution; and (iii) isolates from a natural environment (Fig. 3).

In the Materials and Methods section, the process of comparing and shortlisting candidate environmental strains is described. The two shortlisted environmental isolates (i.e., e1, e6) were found to yield lower urease activity than the banked microorganisms; however, their activities remained comparable to those of the isolates from the continuous growth in the 900 L reactors. Both the environmental isolates and the isolates from the large-scale reactor were identified as *S. pasteurii* by sequencing and database matching, yielding an identity percentage of 85%. The characteristics of the isolates and their urease activity values are reported in Table 1.

**Table 1:** List of the isolates collected and characterized. The same letters in the conductivity kinetics columns indicate significant differences at *P* < 0.05 according to one-way ANOVA and Tukey’s post hoc analyses.

ID	Source	Safety	Urease activity U/L	Conductivity kinetics			
				Rate [mS/cm h]		Value at plateau	
wt	ATCC11859	yes	3085	0.27	a	68.15	a
nhaC	CCOS 1977	yes	2031	0.32	a	68.26	a
p1	Process enrichment	yes	2504	0.12	b	61.25	b
p3		yes	1592	0.06	b	29.83	e
b1	Booster solution	yes	2746	0.11	b	53.39	c
b2		yes	2162	0.10	b	55.29	c
e1	Environment	yes	235	0.05	b	38.81	d
e10		yes	554				
e6		yes	1296	0.11	b	60.94	b
e2		no	na				
e8		no	na				
e10		no	na				
e12		no	na				

**Fig. 4:**
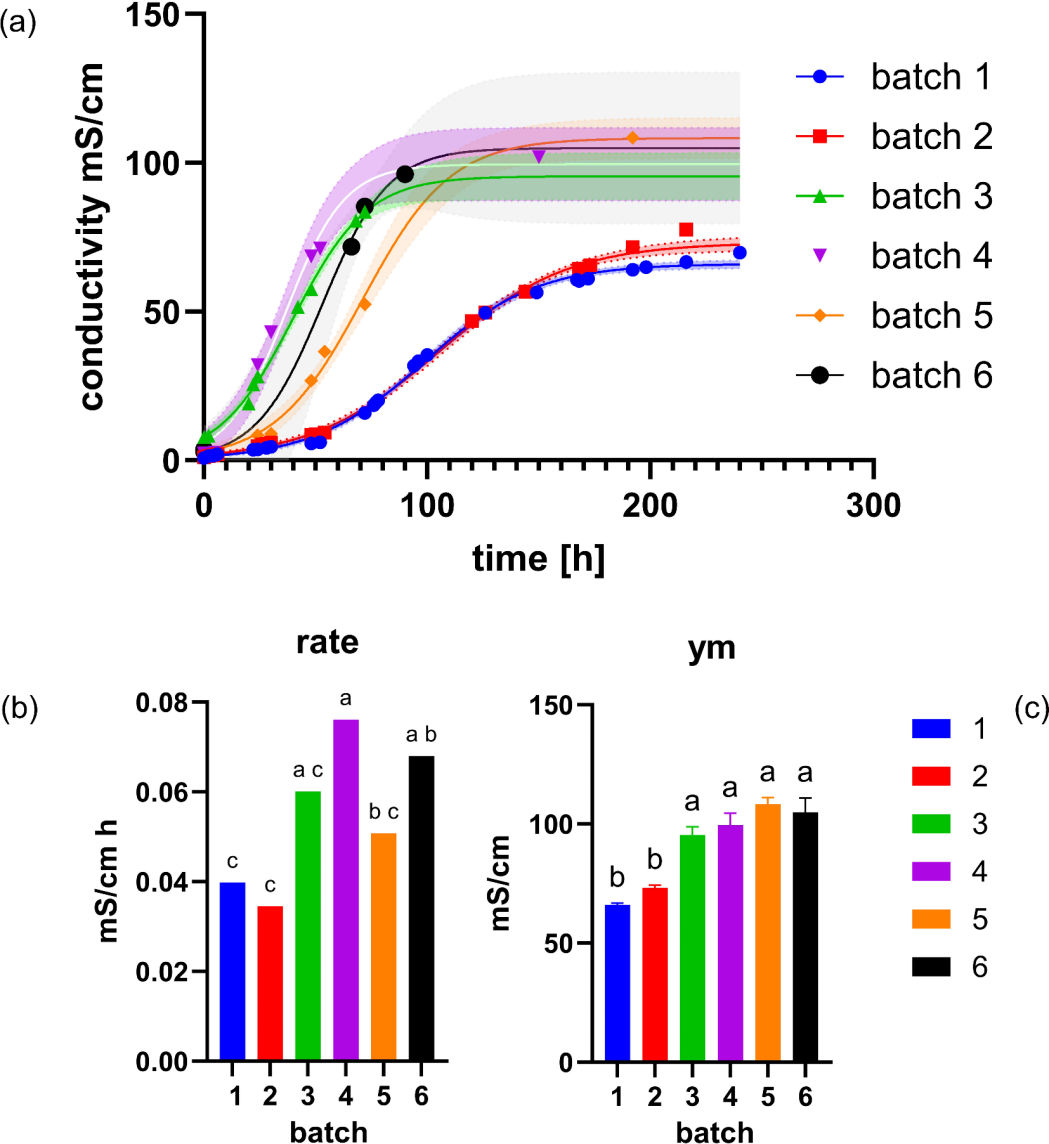
Bioreactor-scale analysis of the growth of ureolytic species in 900 L batches. The same letters in the conductivity kinetics columns indicate significant differences at *P* < 0.05 according to one-way ANOVA and Tukey’s post hoc analyses.

When conductivity was used as a proxy for ion content (NH_4_^+^)^32^, in our analyses, the best performing strain (nhaC) reached a plateau at approximately 70 mS/cm (Fig. 4). Raw data for conductivity, cell counts and urease are reported in the supplementary material (S1). We postulate that the difference between the measured conductivity plateau and the theoretical maximum value is attributed to partitioning in the gas phase. The conductivity evolution of the six separate cultures was investigated by assessing the following kinetic parameters: the maximum slope of the curve (r_m_) and the maximum value at plateau (y_m_) (Fig. 3 b, c). The best performing strains were the *S.pasteurii* wt and the nhaC isolate: both are in fact faster at hydrolyzing urease with the fastest release of H + ions, which corresponds to a rapidly increasing EC. Based on these results, the nhaC strain was chosen to grow in 900 L reactors for a total of six batches and for the subsequent production of a biocemented column.

Batches of 900 L are produced using the upscaled MICP production setup described in the Materials and Methods section. In total, six batches are produced, and the results are presented from the first (#1) to the last batch (#6). Batches #3 to #6 used a residual 50 L from the previous batch each. This implies that the first two batches rely solely on inoculum from the 8 L cultures, i.e., the booster step described in the Materials & Methods, whereas the following batches receive both the 8 L booster step and the 50 L substrate of the preceding batch.

The results revealed relatively slower starts for the first and second batches, which both resulted in steady-state conditions in terms of ECs of approximately 60–65 mS/cm, consistent with the laboratory-bench scale campaign presented in Fig. 3. However, maintaining a fresh inoculum of 50 L in the reactor to launch the next batch significantly improved the hydrolysis of the batch. The results shown in Fig. 4 were used to compare the maximum slope of the hydrolysis curve (r_m_) and the maximum value at plateau (y_m_).

## Assessment of MICP in a large-scale column prototype

**Fig. 5:**
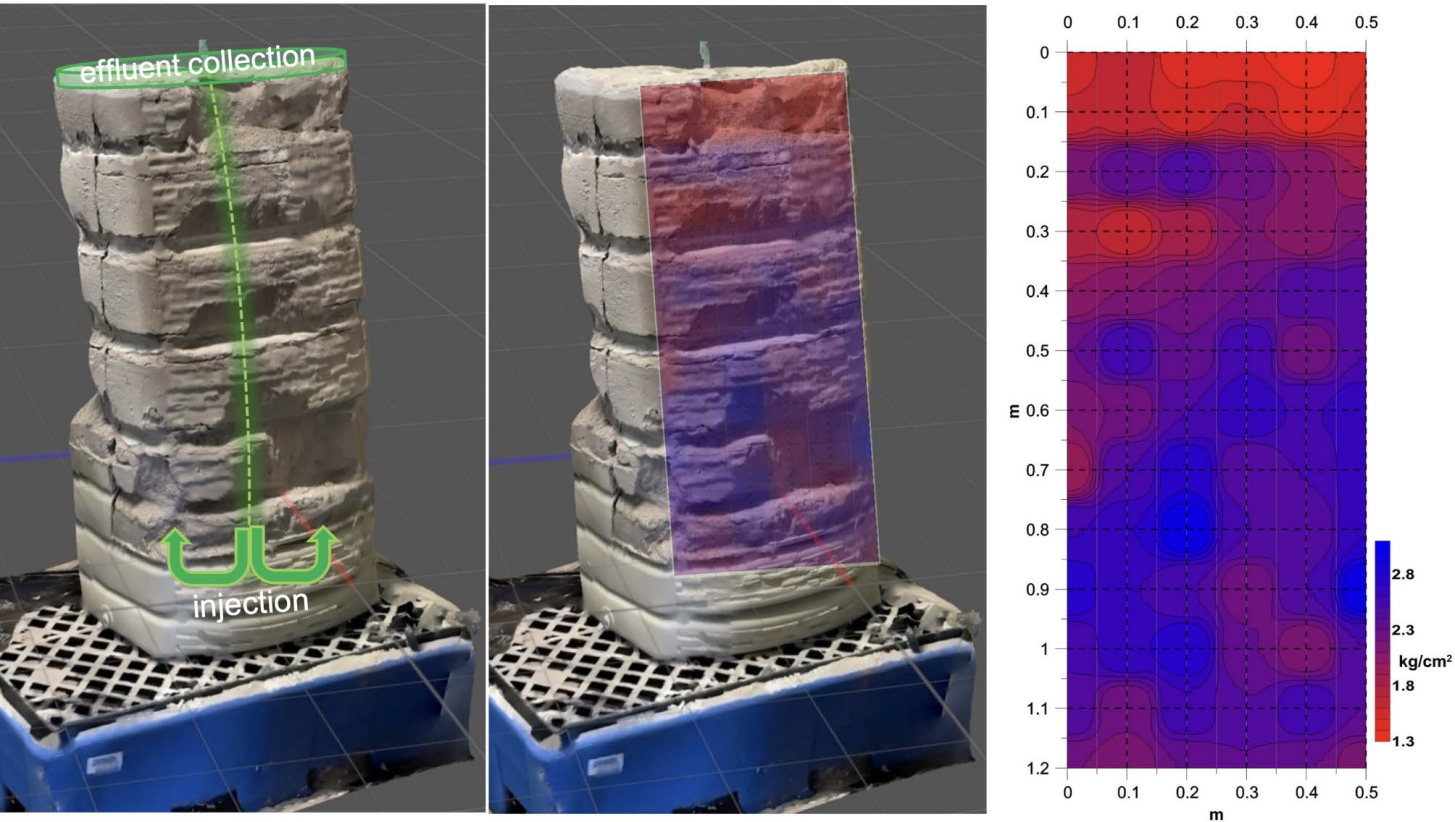
A sand column composed of 0–1 mm aggregates infiltrated via a 25 mm tube was used to apply a bottom-to-top flow path at 5 L/min. The resulting spectrum of strength (kg/cm^2^) from the surface of the column was assessed using a pocket penetrometer.

The last cultured batch from the aforementioned series was then used to infiltrate a column with a height of 1.5 m and diameter of 0.5 m, followed by the continuous circulation of a urea-CaCl_2_ solution. The results revealed that after the removal of the top 1.2 m of the casing, the sand column remained largely intact in the absence of confinement, which was the result of biocementation. The strength distribution on the front face of the column was evaluated using a pocket penetrometer, following a 10 × 10 cm grid pattern (Fig. 5). Similar methods have been used in model experiments using MICP, as described in similar studies^33^. With the exception of the top 15 cm— farthest from the infiltration outlet and effluent collection point—the remainder of the column presented strength values ranging from 1.8 to 2.8 kg/cm². The use of an empirical correlation commonly used^34^ to estimate the undrained Tresca strength from the pocket penetrometer resistance yields values between 90 and140 kPa.

## Conclusions

This work presents findings across various scales concerning the application of MICP. Starting from the very source of the metabolic pathway implicated in MICP, i.e., ureolytic microorganisms, the work sheds light on the ureolytic performance of naturally occurring microorganisms. Specifically, at the bench scale, eight species from three different sources—(i) microorganism banks; (ii) isolates from the continuous growth of the former in nonsterile, large-scale bioreactors; and (iii) from a natural environment—are analyzed. Here, the banked strains were the most prevalent. The continuous growth of the selected strain in the 900 L batches revealed a ureolysis process that reached a plateau in approximately 100 h. No sign of contamination was identified, as all strains present in the bioreactor after continuous growth were *Sporosarcina pasteurii*. The produced bacterial medium was used to infiltrate a sand column with a height of 1.5 m, followed by 3 cycles of biocementation. After 24 h of infiltration, the produced column is endowed with sufficient strength to sustain its weight and remain integrated in the absence of confinement. The analysis of the front façade of the column reveals undrained Tresca strength values between 90 and 140 kPa, which is sufficient to serve a variety of geotechnical and building applications. Overall, this work aims to bridge the knowledge obtained across different scales and establish a shift in the paradigm toward the large-scale application of MICP to address practical problems, introducing notions of scalability and circularity through the treatment of unwanted, ammonia-rich byproducts.

## Electronic supplementary material

Below is the link to the electronic supplementary material.

Supplementary Material 1

## Data Availability

The data supporting the findings of this study are openly available and can be accessed upon reasonable request. Interested readers are encouraged to contact the corresponding author (dimitrios.terzis@epfl.ch) for further details.
