# Supplementary material for: Biocementation beyond the Petri dish, scaling up to 900 L batches and a meter-scale column
Source: Sci Rep. 2025 Jan 24;15:3030. doi: 10.1038/s41598-025-87074-9 (PMC11761496; doi:10.1038/s41598-025-87074-9)
Supplement: Supplementary file 1 — Supplementary Material 1 [file 41598_2025_87074_MOESM1_ESM.docx]

Supplementary material

Cell counts were performed using flow cytometry (Accuri C6 plus, BD, USA) following the Live-Dead protocol (Microbial Viability Measurements on the BD Accuri™ C6 Flow Cytometer). The data for the nhaC isolates are reported in Table S1.

Table S1: nhaC strain growth characterization in terms of cell count, conductivity and urease activity

| Time | Cell counts | Conductivity | Urease activity |
| --- | --- | --- | --- |
| [h] | [log cell n/mL] | [mS/cm] | [U/L] |
| 0 | 0.00 | 1.62 | 0.00 |
| 1 | 4.89 | 1.75 | 16.06 |
| 2 | 5.44 | 2.21 | 38.87 |
| 4.5 | 5.68 | 4.89 | 211.02 |
| 6 | 6.07 | 8.96 | Nd |
| 7 | 6.85 | 15.49 | 721.79 |
| 9 | 8.12 | 29.60 | 854.87 |
| 11.5 | 8.14 | 44.40 | Nd |
| 24 | 8.30 | 49.90 | 30653.72 |
| 27 | 8.37 | 48.10 | 29999.87 |
| 29.5 | 8.36 | 47.70 | Nd |
| 31 | 8.41 | 46.20 | Nd |
| 37.5 | 7.89 | 45.70 | 28846.02 |
| 47 | 9.30 | 46.20 | 11730.64 |
| 51.5 | 9.31 | 45.40 | 14653.72 |

A Spearman correlation coefficient of 0.84 was obtained between conductivity and cell number.
